# Serum vitamins and homocysteine levels in autoimmune liver disease: A systematic review and meta‐analysis

**DOI:** 10.1002/iid3.1258

**Published:** 2024-04-23

**Authors:** Jiahuan Li, Shan Tian, Bai Ci, Yuwen Xi, Xiaoling Deng

**Affiliations:** ^1^ Department of Infectious Diseases, Union Hospital, Tongji Medical College Huazhong University of Science and Technology Wuhan China; ^2^ Division of Gastroenterology, Union Hospital, Tongji Medical College Huazhong University of Science and Technology Wuhan China

**Keywords:** autoimmune hepatitis, autoimmune liver disease, meta‐analysis, primary biliary cholangitis, vitamins

## Abstract

**Objective:**

Vitamins and homocysteine (Hcy) are involved in liver metabolism and related to the pathogenesis of autoimmune liver disease (AILD), but consensus is lacking. This study aims to systematically summarize relevant evidence to clarify the association of serum vitamins and Hcy levels with AILD.

**Methods:**

The English and Chinese literature was searched until August 29, 2023. Studies were included if they were observational studies of investigating serum vitamins and Hcy levels in patients with AILD and their healthy comparisons. Quality assessment was performed by using the Newcastle–Ottawa Scale, and a meta‐analysis was conducted using ReviewManager 5.3. The protocol was registered in the international prospective register of systematic reviews (PROSPERO), with registration number CRD42023455367.

**Results:**

A total of 25 case–control studies comprising 3487 patients (1673 patients and 1814 healthy controls) were included for analysis. There were 548 autoimmune hepatitis (AIH) cases, 1106 primary biliary cholangitis (PBC) cases, and 19 primary sclerosing cholangitis (PSC) cases. We found that serum A and E were decreased in both AIH and PBC/PSC; but vitamin C was reduced only in patients with PBC, not AIH. In addition, decreased content of 25(OH)D3 was found in both AIH and PBC. However, levels of 25(OH)D did not differ between the patients and controls, and were independent of disease types and the country. Only one study that met the inclusion criteria reported vitamin B6, B9, B12, and Hcy changes, and found that vitamin B6 and B9 were significantly decreased in patients with PBC, while serum vitamin B12 and Hcy levels were significantly elevated in them. One eligible study each confirmed a reduction in plasma vitamin K1 and 1,25(OH)2D3 in patients with PBC.

**Conclusion:**

Most vitamins are deficient in AILD, so appropriate vitamin supplementation should be necessary. Further studies with larger sample sizes are needed to validate these findings.

## Graphical abstract

1. Serum vitamin A, E, and 25(OH)D3 were decreased in both autoimmune hepatitis (AIH) and primary biliary cholangitis (PBC)/primary sclerosing cholangitis.
2. Serum vitamin C was reduced only in patients with PBC but not in AIH.
3. Most vitamins are deficient in autoimmune liver disease, and it is necessary to supplement vitamins in moderation.

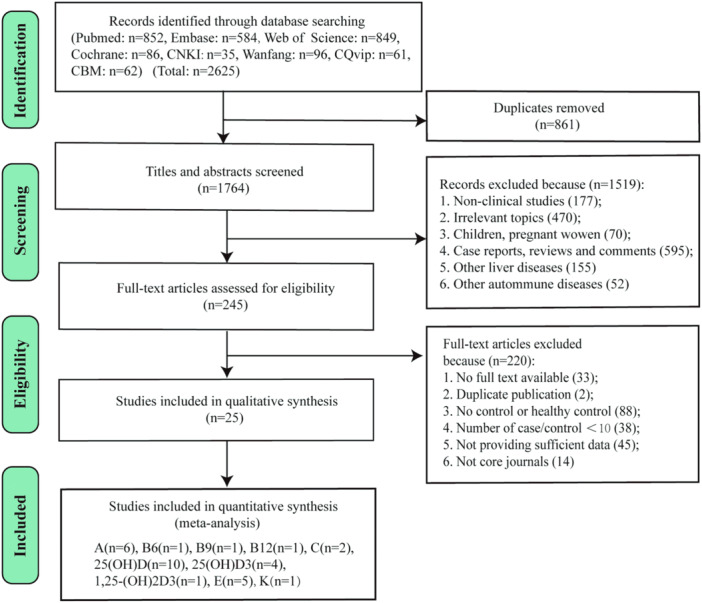

## 1 INTRODUCTION

Autoimmune liver disease (AILD) is a common type of potentially life‐threatening chronic liver disease with autoimmune etiology, which is characterized serologically by elevated transaminases, gammaglobulinemia, and specific autoantibodies, and histologically by interfce heptitis, massive lymphoid and plasma cells infiltration in the portal tract and lobules, as well as sometimes hepatic rosette formation and emperipolesis.^1^ Based on histopathological features and differences in autoantibody expression, AILD can be classified as autoimmune hepatitis (AIH), primary biliary cholangitis (PBC), primary sclerosing cholangitis (PSC), and overlap syndrome. AILD may initially be asymptomatic. With the progression of the disease, it can develop into liver fibrosis, cirrhosis, even acute fulminant hepatitis or end‐stage liver disease.^2^ Some cryptogenic hepatocellular carcinoma may also be attributed to ALID.^2^ Currently, AILD is increasingly occurring with contemporary global incidence per 100,000 for PBC ranging from 0.84 to 2.75, for PSC between 0.1 and 4.39, and for AIH from 0.4 to 2.39. And AILD accounted for 24% of liver transplantation cases in Europe and the United States.^3^ But, up to 40% of AILDs inevitably recur after liver transplantation despite preoperative immunosuppressive therapy.^4^, ^5^ In addition, AILD is often accompanied by autoimmune diseases of other systems, like inflammatory bowel disease, Sjogren's syndrome.^6^, ^7^ Therefore, AILD has become a serious chronic liver disease, causing huge economic burden to the society.^4^, ^5^, ^8^

AILD may occur due to genetic predisposition, environmental factors (smoking, drug, and xenobiotic exposure, various hepatitis virus infections), loss of immune tolerance to self‐antigens, immune disorders, as well as alterations of commensal intestinal flora.^9^, ^10^ But, the exact etiology and pathogenesis of AILD is still unclear. Recently, there is a certain amount of studies reporting vitamin deficiency in PBC or AIH.^11^, ^12^, ^13^ Since vitamins are trace organic substances necessary to maintain normal physiological functions of the body, thus vitamins deficiency may play a prominent role in the development of AILD.

Each vitamin has a unique role in liver disease. Vitamin A, in the form of retinol, is mainly stored in hepatocyte and hepatic stellate cells (HSC), especially in HSC lipid droplets.^14^ It has been reported that the rate of vitamin A deficiency in chronic liver disease is as high as 62.4%, and the degree of serum retinol deficiency was positively correlated with the severity and progression of liver disease.^12^ B vitamins are a kind of water‐soluble small molecule compounds, which are widely involved in various physiological processes in the form of co‐enzymes. These following B vitamins may be associated with AILD: B6, B9 (folic acid), and B12, which work together to participate in the folic acid cycle and methionine metabolism, and promote the production and maturation of red blood cells. Deficiencies such as vitamin B9, vitamin B6, or vitamin B12 can cause hyperhomocysteine.^15^, ^16^ Homocysteine (Hcy) is shown to be decreased in a variety of liver diseases.^17^, ^18^

Vitamin C, also known as ascorbic acid, is a powerful antioxidant with the ability to scavenge many physiological free radicals, promote iron absorption, detoxification, etc.^19^ Compared with healthy controls, serum vitamin C levels were found significantly decreased in PBC patients,^20^ but did not change in AIH patients, as demonstrated in another study.^21^

Vitamin D is a class of important fat‐soluble ring‐opening sterol, which main functions include the regulation of blood calcium and phosphorus concentration, new bone formation and calcification, promotion of the growth and differentiation of skin cells, and immune function modulation.^22^ Vitamin D deficiency in patients with chronic liver disease has been reported in a number of studies.^11^, ^23^, ^24^ It has been indicated that severe vitamin D deficiency can be an important prognostic biomarker in AIH or PBC, which is closely associated with incomplete response to ursodeoxycholic acid, progression to cirrhosis, and liver‐related death or even need for liver transplantation.^24^, ^25^ However, some studies have found no change or even a slight increase in vitamin D levels in patients with AILD.

Vitamin E is also an indispensable fat‐soluble multivitamin and exerts strong antioxidant and antiaging ability.^26^ For AILD, most studies have reported lower serum vitamin E levels in patients.^21^, ^27^, ^28^ But some research works have found no difference in vitamin E levels between AILD patients and healthy counterparts.^20^

Vitamin K is an important fat‐soluble vitamin in the body and plays a critical role in the detoxification of xenobiotics, synthesis of coagulation factors, and metabolism of bile acid (BA).^29^ As a coenzyme of γ‐hydroxylase, vitamin K participates in the synthesis of coagulation factors II., VII., IX., X., anticoagulant protein C, and anticoagulant S in the liver.^30^ Thus, vitamin K deficiency is common in different forms of liver disease, including cholestasis, and is significantly associated with the severity of liver disease, as demonstrated by prolonged prothrombin time (PT).^30^, ^31^ However, the use of vitamin K supplementation for the treatment of patients with liver disease is somewhat controversial.^32^, ^33^

Due to the above controversy, there is no consensus on whether vitamin supplements should be taken, or what type of AILD should be treated with vitamin. In addition, few meta‐analyses or systematic review studies have been reported on the effects of serum vitamins and Hcy changes on AILD. Thus, we systematically summarized relevant evidence and conducted this meta‐analysis to clarify the association between serum vitamins and Hcy levels with AILD, with a view to contributing to the clinical prevention and treatment of AILD.

## 2 MATERIALS AND METHODS

### 2.1 Search strategy

This meta‐analysis was performed in strict accordance with the preferred reporting items for systematic reviews and meta‐analyses (PRISMA) statement.^34^ Eight commonly used Chinese and English electronic databases were searched for available studies up to August 29, 2023 with no language restriction: PubMed, Embase (including conference abstracts), the Cochrane Library, Core collection in Web of Science, Chinese National Knowledge Infrastructure (CNKI), Chinese Biological Medicine (CBM), Wanfang and CQVIP databases. We established the following search strategy in PubMed: ((“autoimmune liver disease”[Mesh]) OR “autoimmune hepatitis” OR “AIH” OR “primary biliary cholangitis” OR “primary biliary cirrhosis” OR “PBC” OR “primary sclerosing cholangitis” OR “PSC” OR “autoimmune cholangitis” OR “autoimmune sclerosing cholangitis”) AND (“vitamin” OR “vitamin A” OR “retinol” OR “retinoic acid” OR “B‐vitamins” OR “vitamin B complex” OR “vitamin B6” OR “pyridoxamine” OR “pyridoxal” OR “vitamin B9” OR “folic acid” OR “folate” OR “folic acid, (DL)‐Isomer” OR “cyanocobalamin” OR “cobalamin” OR “vitamin B12” OR “ascorbic acid” OR “vitamin C” OR “vitamin D” OR “1,25(OH)(2) vitamin D”OR “25(OH) vitamin D” OR “25(OH) vitamin D3” OR “ergocalciferol” OR “calcitriol” OR “vitamin E” OR “tocopherols” OR “vitamin K” OR “vitamin K1” OR “phylloquinone” OR “vitamin K2” OR “menaquinone”). The same retrieval strategy was also applied in other databases. In addition, the reference lists of the relevant studies were reviewed to avoid missing any publication.

### 2.2 Selection criteria

In this step, two authors (JH Li and S Tian) independently assessed the potentially eligible studies. The following inclusion criteria were used in this meta‐analysis: (1) observational studies of human beings diagnosed with AILD; (2) patients in the control group were matched healthy comparisons; (3) studies providing sufficient data about serum vitamin (A, B, C, D, E, K, etc.) and Hcy levels. The exclusion criteria were: (1) adolescents (under 18 years of age) and pregnant women; (2) studies with less than 10 patients in the control group or the case group; (3) animal and cell experiments; (4) reviews, comments, case reports, expert consensus, duplicated reports, studies with incomplete data and no full text available; (5) other liver diseases: including liver transplantation, liver cancer, fatty liver, alcoholic liver disease, etc.; (6) other autoimmune diseases: autoimmune atrophic gastritis, inflammatory bowel disease, and so forth; (7) articles published in the non‐Chinese core journals. Any disagreements regarding the inclusion or exclusion of the articles were resolved by consensus or adjudicated by the third reviewer (XL Deng).

### 2.3 Quality assessment

Quality assessment of all studies was conducted by two independent reviewers (B Ci and YW Xi) using the Newcastle–Ottawa Scale.^35^ In brief, each included study was evaluated from three aspects (selection, comparability control for important factors, and exposure), with a total of 9 stars. Studies with six or more stars are considered high quality. Any discrepancies regarding the quality assessment were discussed or resolved by another experienced researcher (XL Deng).

### 2.4 Data extraction

Two investigators (JH Li and S Tian) independently extracted available data from each study and double‐checked all information. The following details were recorded: authors, year of publication, country, sample sizes of case/control groups, patient gender and mean age, research type, method of assay, type of AILD (AIH, PBC, or PSC), type and levels of vitamins and Hcy in the patient serum.

### 2.5 Statistical analysis

The ReviewManager 5.3 software was used to analyze differences in serum vitamin and Hcy levels between healthy controls and patients with AILD. Subgroup analyses were performed based on the type of AILD (AIH, PBC, and PSC), country (China and other countries). The mean differences (MD) with 95% confidence intervals (CIs) were calculated for continuous variables with uniform measurement methods and unit, otherwise, standardized mean difference (SMD) was selected. Differences of *p* < .05 were accepted as significant. Fixed effects models or random effects models were conducted for low heterogeneity (*I* ^2^ < 50%) or significant heterogeneity (*I* ^2^ ≥ 50%) across the studies, respectively. When the number of papers was greater than or equal to 10, funnel plot was used to detect publication bias. Sensitivity analysis was used to test the impact of individual studies by Stata 15.1 software (StataCorp).

### 2.6 Register name and registration number

The protocol was registered in the international prospective register of systematic reviews (PROSPERO) by JH Li, with registration number CRD42023455367. Our meta‐analysis is consistent with the registration information.

## 3 RESULTS

### 3.1 Characteristics of the enrolled studies

**Figure 1:**
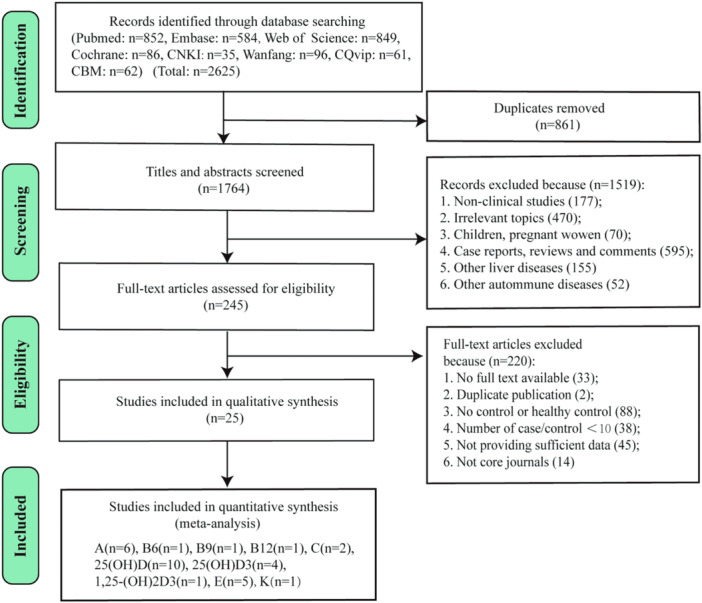
PRISMA flow diagram of the study selection process.

As shown in Figure 1, a total of 2625 records were originally obtained from eight databases. After removing 861 duplicates, 1519 records were excluded in turn by screening the titles and abstracts for the following reasons: no‐clinical studies (n = 177); irrelevant topics (*n* = 470); children and pregnant women (*n* = 70); case reports, reviews and comments (*n* = 595); other liver diseases (*n* = 155); and other autoimmune diseases (*n* = 52). Then, 245 full‐text articles were carefully reviewed for eligibility, and 220 of these were subsequently excluded for no full text available (*n* = 33), not core journals (*n* = 14), duplicate publication (*n* = 2), number of cases or controls < 10 (*n* = 38), no control or no healthy control (*n* = 88), and not providing sufficient data (*n* = 45). Ultimately, 25 studies^11^, ^20^, ^21^, ^23^, ^24^, ^27^, ^28^, ^36^, ^37^, ^38^, ^39^, ^40^, ^41^, ^42^, ^43^, ^44^, ^45^, ^46^, ^47^, ^48^, ^49^, ^50^, ^51^, ^52^, ^53^ with a total of 3487 patients (1673 patients, 1814 healthy controls) were enrolled in the meta‐analysis. All included studies were case–control studies, 10 of which were conducted in China, and the remaining research works were from other countries, including Italy, Israel, Spain, Turkey, England, Sweden, UK, and USA. There were 548 AIH cases, 1106 PBC cases, and 19 PSC cases. Most cases were female. The detailed characteristics of all studies are summarized in Table 1. The detection methods of vitamins vary widely, including radioimmunoassay (RIA), high‐pressure liquid chromatography (HPLC), chemical luminescence immunoassay (CLIA), enzyme‐linked immunosorbent assay (ELISA), liquid chromatography‐tandem mass spectrometry, and microfluorometric method. The changes of serum vitamins and Hcy levels per study are depicted in Table 2.

**Table 1:** Characteristics of included studies in the meta‐analysis.

Study	Country	Research type	Disease type	Method of assay	Sample size (Case/Control)	Age (year)		Gender (male/female)		Observation index	Quality assessment
						Case	Control	Case	Control		
Huang and Hu^36^	China	Case–control	AIH	ELISA	187/150	52.37 ± 8.04	53.42 ± 8.11	52/135	27/123	25‐(OH)D3	8
Wang et al.^11^	China	Case–control	PBC	CLIA	185/141	56.06 ± 11.48	54.41 ± 10.04	15/170	18/123	25(OH)D3	8
Sen et al.^23^	China	Case–control	AIH	Liquid chromatography‐tandem mass spectrometry	80/80	NA	NA	NA	NA	25‐(OH)D	6
Li et al.^37^	China	Case–control	PBC	CLIA	74/66	59.4 ± 6.8	57.1 ± 5.3	32/42	31/35	25‐(OH)D	8
Lin et al.^38^	China	Case–control	PBC	Electrochemiluminescence immunoassay	67/50	52.66 ± 11.47	46.37 ± 10.20	8/59	21/29	25‐(OH)D	8
Zhang^39^	China	Case–control	PBC	ELISA	80/80	56.74 ± 6.83	55.65 ± 5.81	12/68	NA	25‐(OH)D	8
Tian and Wang^40^	China	Case–control	AIH	CLIA	30/18	31.4 ± 22.5	36.4 ± 23.1	17/13	9/9	25‐(OH)D3	8
Li and Song^41^	China	Case–control	AIH	ELISA	150/70	47.4 ± 11.9	45.5 ± 11.1	14/136	10/60	25‐(OH)D	8
Yang et al.^42^	China	Case–control	PBC	HPLC	17/300	54.29 ± 13.29	50.0 ± 11.0	3/14	185/115	25‐(OH)D	7
Agmon‐Levin et al.^24^	Italy, Israel and Spain	Case–control	PBC	CLIA	79/70	NA	NA	NA	NA	25‐(OH)D	8
Efe et al.^43^	Turkey	Case–control	AIH	Liquid chromatography‐mass spectrometry	68/34	41.9 ± 13.1	43.8 ± 11.1	19/49	7/27	25‐(OH)D3	8
Cash et al.^20^	England	Case–control	PBC	HPLC	51/34	56.2 ± 10.8	54.0 ± 12.2	2/49	7/27	Vitamin C, Vitamin A, Vitamin E	8
Zhang et al.^44^	China	Case–control	PBC	RIA	10/10	47–58	NA	NA	NA	1,25‐(OH)2D3	7
Biagini^45^	Italy	Case–control	PBC	RIA, HPLC, and fluorescence detecting	51/102	63 ± 13.9	63 ± 13	8/43	16/86	Folic Acid, Vitamin B12, Vitamin B6，Homocysteine	8
Floreani^46^	Italy	Case–control	PBC	RIA	35/33	52.5 ± 10	51.8 ± 2.22	0/35	0/33	25‐(OH)D	7
Pemberton et al.^21^	England	Case–control	AIH	Enhanced chemiluminescent technique	33/35	46.5 ± 17.2	52.2 ± 12.9	5/28	7/28	Vitamin A, Vitamin C, Vitamin E	6
Aboutwerat et al.^47^	England	Case–control	PBC	Enhanced chemiluminescent technique	41/34	57.9 ± 9.0	53.1 ± 11.9	4/37	7/27	Vitamin A, Vitamin E	8
Verma et al.^48^	England	Case–control	PBC	RIA	37/21	60 (45–78)	58 (36–74)	0/37	0/21	25‐(OH)D	8
Floreani et al.^28^	Italy	Case–control	PBC/PSC	HPLC	105/105	51.8 ± 13.5	51.35 ± 10	17/88	17/88	Vitamin A, Vitamin E	8
Kowdley et al.^53^	USA	Case–control	PBC	HPLC	77/255	51 ± 1.1	NA	3/74	NA	Vitamin K1	6
Janczewska et al.^49^	Sweden	Case–control	PBC	HPLC	14/10	55 ± 6	NA	NA	NA	Vitamin A	6
Nyberg et al.^50^	Sweden	Case–control	PBC	Fluorometry	44/25	NA	NA	7/37	6/19	Vitamin A	8
Jeffrey et al.^27^	UK	Case–control	PBC	Coiorimetrically	80/26	NA	NA	NA	NA	Vitamin E	6
Fonseca et al.^51^	UK	Case–control	PBC	RIA	36/40	58 (49‐70)	20‐60	4/32	20/20	25‐(OH)D	6
Sokol et al.^52^	USA	Case–control	PBC	Microfluorometric method	42/25	49.5 ± 9.1	31.6 ± 3.1	2/40	NA	Vitamin E	6

Abbreviations: AIH, autoimmune hepatitis; CLIA, chemical luminescence immunoassay; ELISA, enzyme‐linked immunosorbent assay; HPLC, high‐pressure liquid chromatography; NA, not available; PBC, primary biliary cholangitis; PSC, primary sclerosing cholangitis; RIA, radioimmunoassay.

**Table 2:** Results of studies included in the meta‐analysis.

Year	A	B6	B9	B12	C	25(OH)D	25(OH)D3	1,25(OH)2D3	E	Hcy	K1
Huang and Hu^36^							↓				
Wang et al.^11^							↓				
Sen et al.^23^						↓					
Li and Zhou^37^						↑					
Lin et al.^38^						↓					
Zhang^39^						↓					
Tian and Wang^40^							↓				
Li and Song^41^						↓					
Yang et al.^42^						↓					
Agmon‐Levin et al.^24^						↓					
Efe et al.^43^							↓				
Cash et al.^20^	↓				↓				N		
Zhang et al.^44^								↓			
Biagini^45^		↓	↓	↑						↑	
Floreani^46^						↑					
Pemberton et al.^21^	↓				N				↓		
Aboutwerat et al.^47^	↓				N				N		
Verma et al.^48^						N					
Floreani et al.^28^	↓								↓		
Kowdley et al.^53^											↓
Janczewska et al.^49^	↓										
Nyberg et al.^50^	↓										
Jeffrey et al.^27^									↓		
Fonseca et al.^51^						N					
Sokol et al.^52^									N		

*Note*: *N* represents no change.

All studies were of high quality, with scores greater than 6 stars. However, none of these studies described the no‐response rate. The quality assessment details for all studies are presented in Supporting Information S1: Table S1.

### 3.2 Changes of serum vitamins and Hcy levels in patients with AILD

#### 3.2.1 Vitamin A

**Figure 2:**
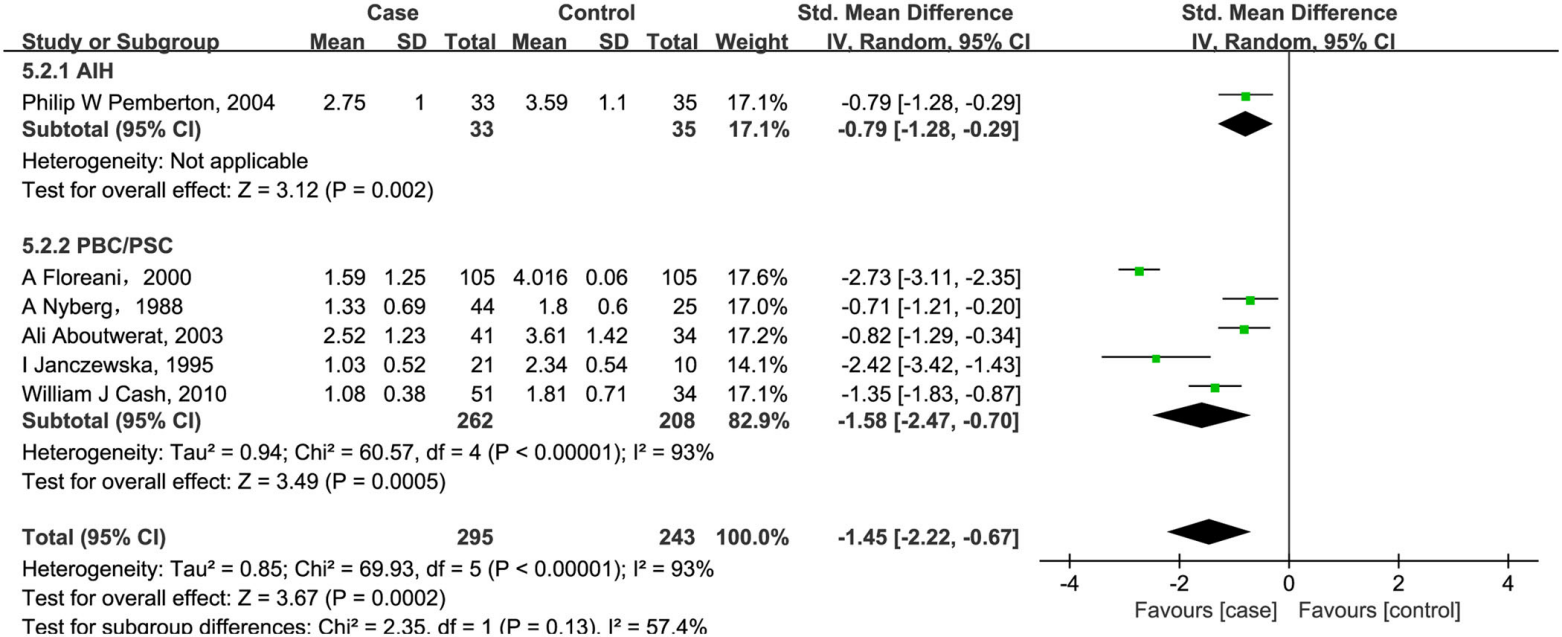
Forest plot of the meta‐analysis on serum vitamin A levels in patients with autoimmune liver disease (AILD). AIH, autoimmune hepatitis; CI, confidence interval; PBC, primary biliary cholangitis; PSC, primary sclerosing cholangitis; SD, standard deviation.

Six included studies^20^, ^21^, ^28^, ^47^, ^49^, ^50^ investigated vitamin A, with 274 patients in case group and 233 healthy individuals in the control group. As shown in Figure 2, vitamin A levels were lower in AILD patients compared with the control group (SMD = −1.45, 95% CI = [−2.22, −0.67], *p* = .0002) with high heterogeneity (Q = 69.93, *I* ^2^ = 93%, *p* < .00001; Figure 2). Further subgroup analysis based on disease types showed that serum vitamin A content was decreased by 1.58 (95% CI = [−2.47, −0.70], *p* = .0005) in PBC/PSC patients. However, heterogeneity is still high across studies (Q = 60.57, *I* ^2^ = 93%, *p* < .00001; Figure 2). Only study^47^ reported serum vitamin A changes in AIH patients, and found that serum A content was decreased by 0.79 in them.

#### 3.2.2 Vitamin B6, B9, B12, and Hcy

Only one study^45^ involving 51 PBC patients and 102 healthy individuals examined vitamin B6, B9, B12, and Hcy together. In the study of Biagini et al.,^45^ compared with the control group, vitamin B6 and B9 were significantly decreased in patients with PBC (vitamin B6: 6.6 [1–20] vs. 10.0 [3–17] pg/mL, *p* < .001; vitamin B9: 5.3 [1.2‐13.4] vs. 10.7 [5.4‐18.5] ng/mL, *p* < .001), while serum vitamin B12 levels were significantly elevated in them (335 [201‐977] vs. 304.9 [176‐427.1] pg/mL, *p* < .05). For Hcy, the content of its fasting state in plasma was also significantly higher in patients than in controls (12.1 ± 8.76 [1.5‐58.8] vs. 9.9 ± 1.7 [6.4‐18.0] µmol/L, *p* < .001).

#### 3.2.3 Vitamin C

**Figure 3:**
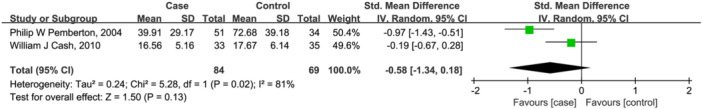
Forest plot of the meta‐analysis on serum vitamin C levels in patients with autoimmune liver disease (AILD). CI, confidence interval; SD, standard deviation.

Two studies^20^, ^21^ evaluated serum vitamin C levels in 84 cases and 69 controls. There is no significant difference between two groups (SMD = −0.58, 95% CI = [−1.34, 0.18], *p* = .13) with high heterogeneity (Q = 5.28, *I* ^2^ = 81%, *p* = .02; Figure 3). Notably, compared with controls, serum vitamin C was decreased in PBC patients, but not in patients with AIH.

#### 3.2.4 25(OH)D, 25(OH)D3, and 1,25(OH)2D3

**Figure 4:**
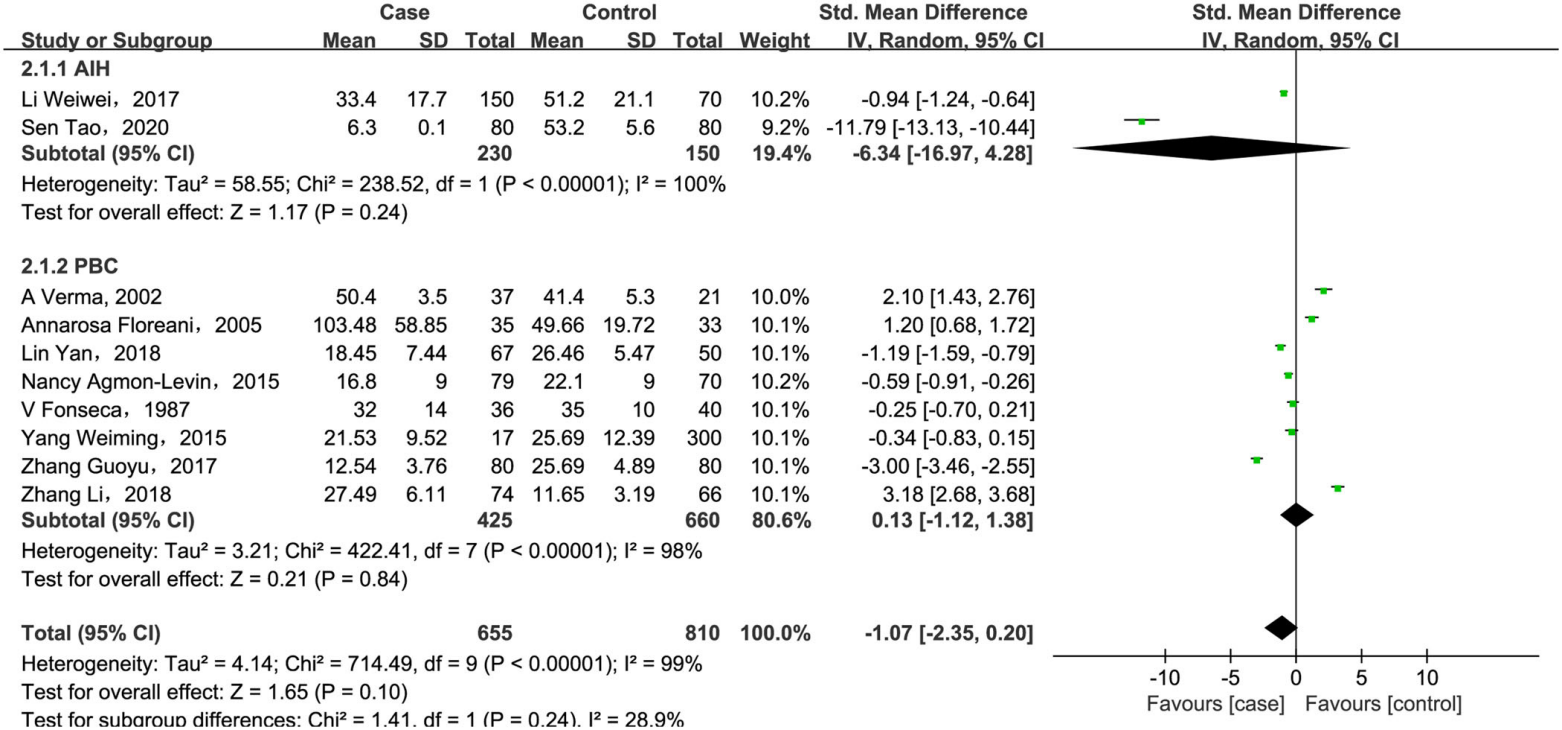
Forest plot of the meta‐analysis on serum vitamin 25(OH)D levels in patients with autoimmune liver disease (AILD). AIH, autoimmune hepatitis; CI, confidence interval; PBC, primary biliary cholangitis; SD, standard deviation.

A total of 10 studies^23^, ^24^, ^37^, ^38^, ^39^, ^41^, ^42^, ^46^, ^48^, ^51^ investigated 25(OH)D (655 case/810 controls), and four research works reported 25(OH)D3^11^, ^36^, ^40^, ^43^ (470 cases/343 controls) changes in AILD patients. For 25(OH)D, there were no significant differences between two groups both in total analysis (SMD = −1.07, 95% CI = [−2.35, 0.20], *p* = .1; Figure 4) and subgroup analysis based on disease types (AIH: SMD = −6.34, 95% CI = [−16.97, 4.28], *p* = .24; PBC: SMD = 0.13, 95% CI = [−1.12, 1.38], *p* = .84; Figure 4). For PBC patients, we further performed a subgroup analysis based on country. But, as shown in Supporting Information S1: Figure S1, no significant changes of 25(OH)D levels were observed both in studies conducted in China (SMD = −0.34, 95% CI = [−2.76, 2.08], *p* = .78) and other countries (SMD = 0.59, 95% CI = [−0.55, 1.73], *p* = .31) in PBC patients. And subgroup analysis also did not reduce heterogeneity between groups, suggesting that confounding factors of unknown origin may be existed.

**Figure 5:**
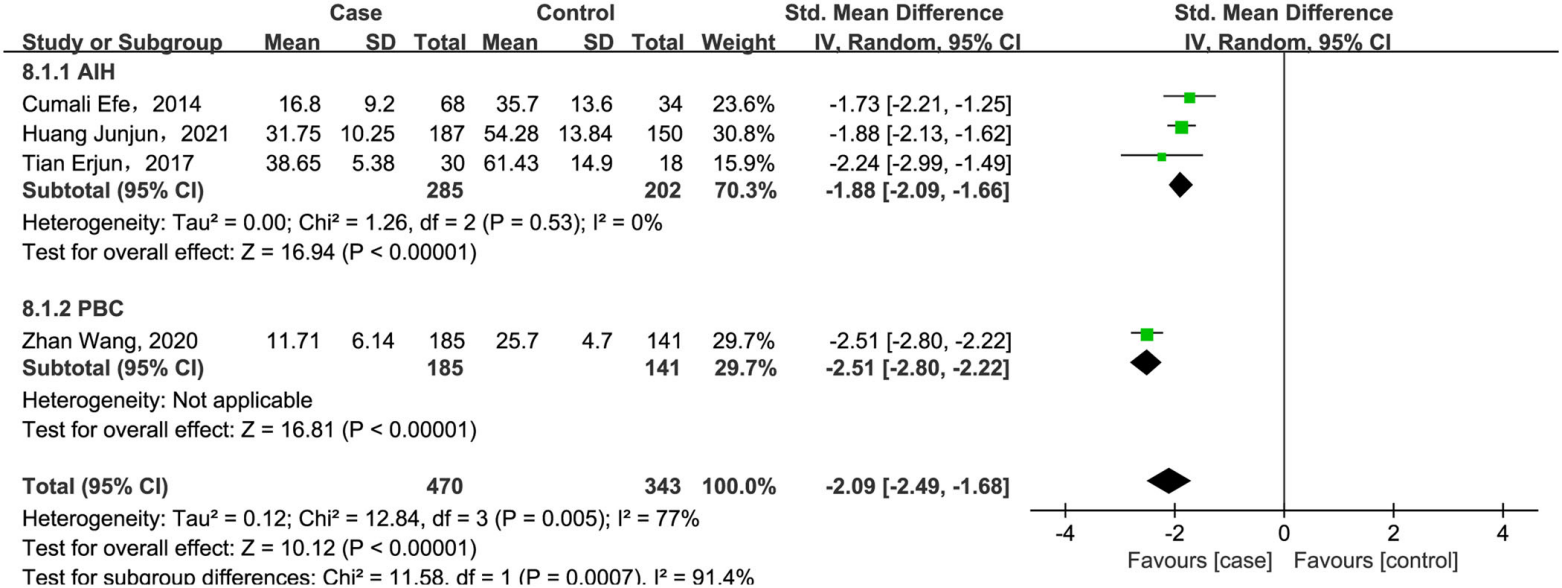
Forest plot of the meta‐analysis on serum vitamin 25(OH)D3 levels in patients with autoimmune liver disease (AILD). AIH, autoimmune hepatitis; CI, confidence interval; PBC, primary biliary cholangitis; SD, standard deviation.

Nevertheless, as shown in Figure 5, compared with healthy controls, 25(OH)D3 levels were significant lower in AILD (SMD = −2.09, 95% CI = [−2.49, −1.68], *p* < .00001) with high heterogeneity (Q = 12.84, *I* ^2^ = 77%, *p* = .005). In addition, 25(OH)D3 levels also decreased in both PBC and AIH (SMD = −1.88, 95% CI = [−2.09, −1.66], *p* < .00001, Figure 5) subgroups, and heterogeneity was totally eliminated after subgroup analysis.

Only one study^44^ reported the serum 1,25(OH)2D3 change in ten PBC patients and equivalent controls and found that 1,25(OH)2D3 level was significant lower in patients with PBC (33.28 ± 4.07 vs. 48.25 ± 5.10 ng/ml, *p* ＜ .01).

#### 3.2.5 Vitamin E

**Figure 6:**
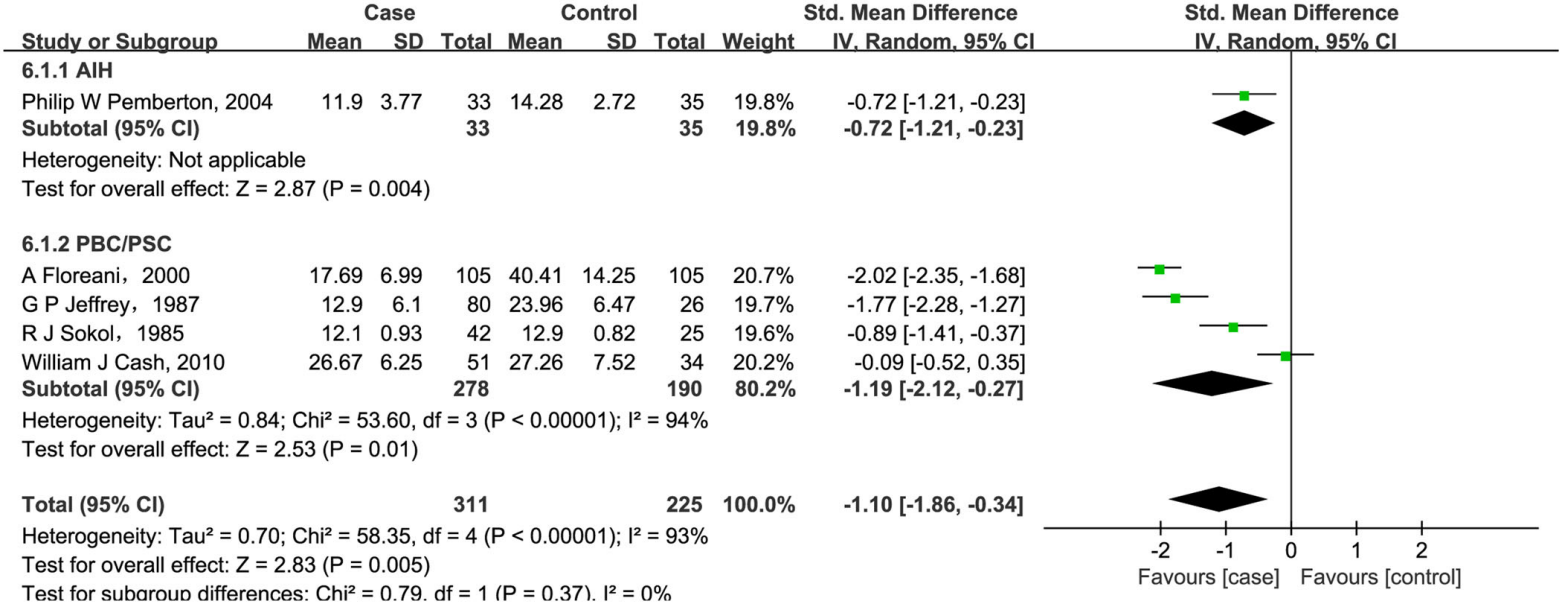
Forest plot of the meta‐analysis on serum vitamin E levels in patients with autoimmune liver disease (AILD). AIH, autoimmune hepatitis; CI, confidence interval; PBC, primary biliary cholangitis; PSC, primary sclerosing cholangitis; SD, standard deviation.

Five studies^20^, ^21^, ^27^, ^28^, ^52^ were for vitamin E (311 cases/225 controls). But, a significant association was found between lower vitamin E level (SMD = −1.01, 95% CI = [−1.86, −0.34], *p* = .005, Figure 6) and AILD. While evidence of heterogeneity was observed with vitamin E (Q = 58.35, *I* ^2^ = 93%, *p* < .00001, Figure 6). Further subgroup analysis based on disease types showed that for PBC/PSC patients, serum vitamin E content decreased by 1.19 (95% CI = [−2.12, −0.27], *p* = .01). Only one study^20^ reported serum vitamin E changes in AIH patients, and found that serum vitamin E content decreased by 0.72 in them. However, heterogeneity was not significantly eliminated despite the subgroup analysis described above.

#### 3.2.6 Vitamin K1

Only one eligible study^53^ discussed the difference between vitamin K1 in 77 PBC patients and 255 healthy controls. It reported that median plasma vitamin K1 level was significantly lower in PBC patients (0.65 [0.05‐4.13] vs. 0.95 [0.2‐ 4.92] nmol/L; *p* < .0001). However, more studies and evidence are needed to verify this conclusion.

### 3.3 Sensitivity analysis

**Figure 7:**
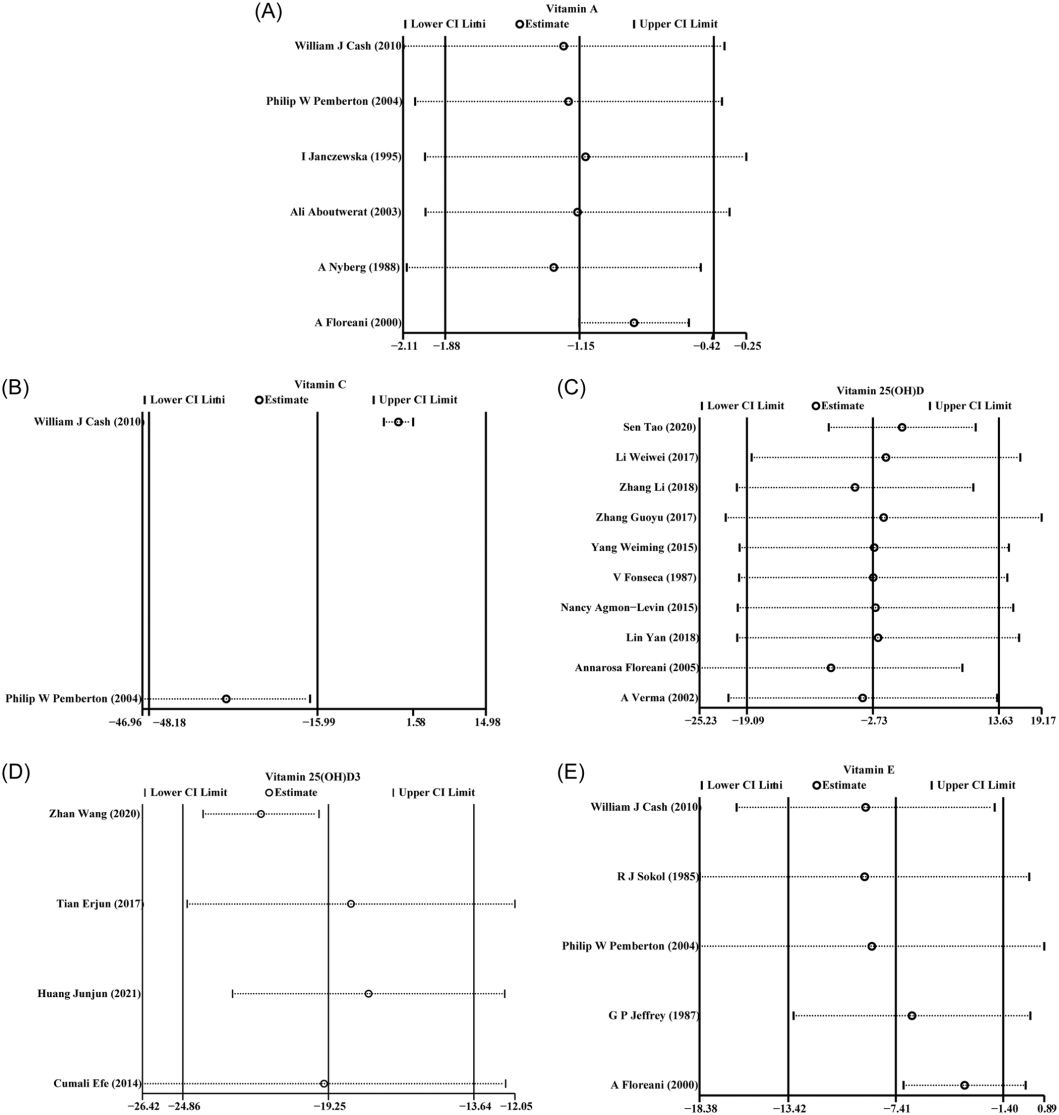
Sensitivity analysis of the meta‐analysis on vitamins levels in patients with autoimmune liver disease (AILD): (A) vitamin A; (B) vitamin C; (C) vitamin 25(OH)D; (D) vitamin 25(OH)D3; (E) vitamin E.

The results of sensitivity analysis demonstrated that the heterogeneity among the included studies did not reverse the overall meta‐analysis results of vitamin A (Figure 7A), vitamin C (Figure 7B), 25(OH)D (Figure 7C), 25(OH)D3 (Figure 7D), and vitamin E (Figure 7E). In addition, there was no obvious influence of one individual study on the pooled SMDs.

### 3.4 Publication bias

**Figure 8:**
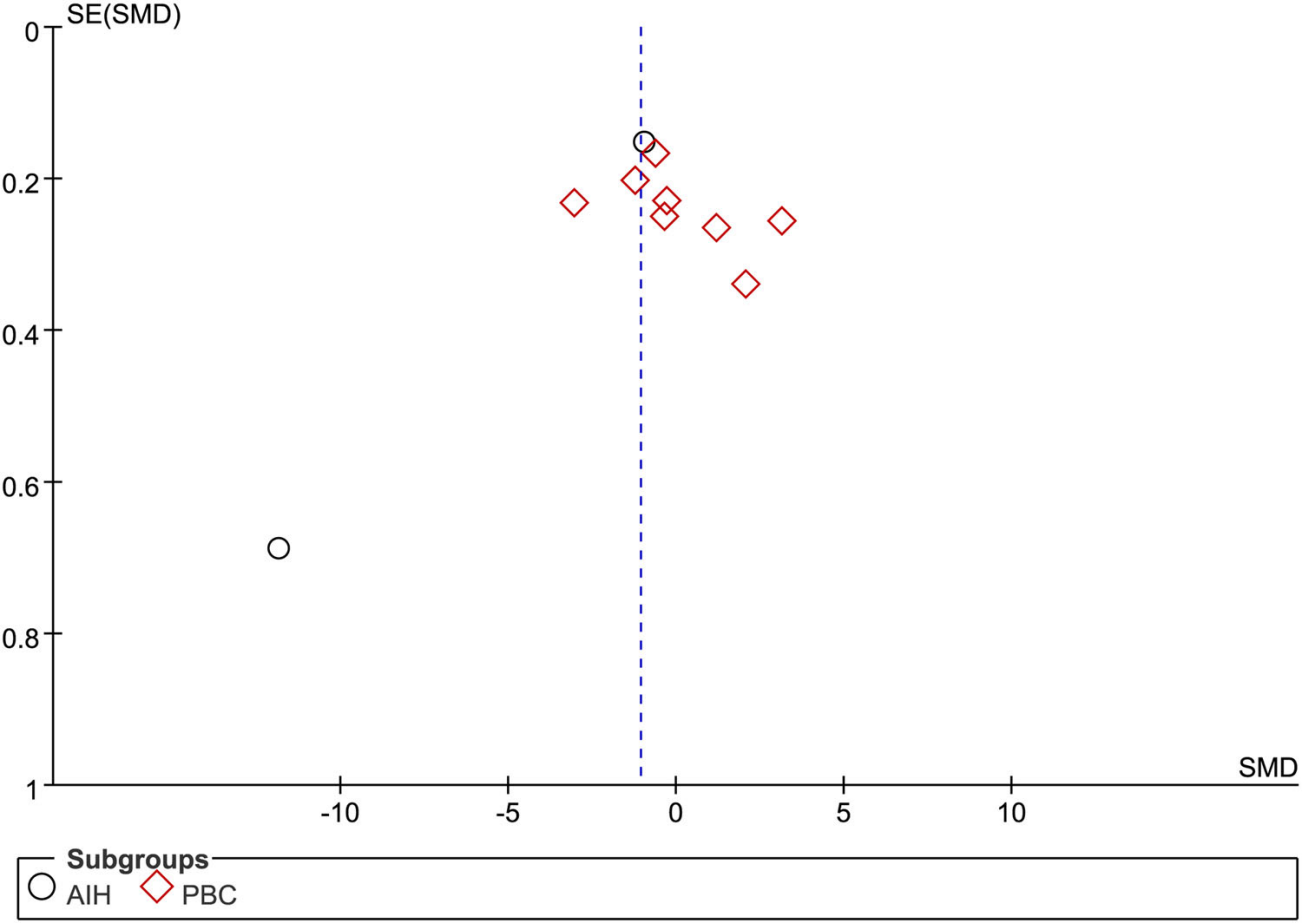
Funnel plot of 10 included studies in this meta‐analysis for serum vitamin 25(OH) D levels in patients with autoimmune liver disease (AILD). AIH, autoimmune hepatitis; PBC, primary biliary cholangitis; SMD, standardized mean difference.

Only the number of studies about 25(OH)D more than 10, thus a funnel plot, Begg's and Egger's tests were employed to investigate publication bias. However, the shape of the funnel plot for this indicator did not seem symmetrical (Figure 8). Notably, Begg's tests (*p* = 1.0) and Egger's tests (*p* = .337) of the meta‐analysis suggested no exsited significant publication bias. These results indicated a potential bias other than publication bias, like the limited involved studies and enrolled patients.

## 4 DISCUSSION

To the best of our knowledge, this is the first meta‐analysis to systematically explore the association of serum vitamins and Hcy levels with AILD. We found that serum A and E were decreased in both AIH and PBC/PSC, but vitamin C was decreased only in patients with PBC, not AIH. In addition, the content of 25(OH)D3 was decreased both in AIH and PBC. However, 25(OH)D is shown no difference between the two and independent of the country. Only one study that met the inclusion criteria reported B vitamins and Hcy changes, and found that vitamin B6 and B9 were significantly decreased in patients with PBC, while serum vitamin B12 and Hcy levels were significantly elevated in them. One eligible study each confirmed a decrease in plasma vitamin K1 and 1,25(OH)2D3 in patients with PBC. Thus, most vitamins seem to be deficient in AILD.

Our meta‐analysis shows that serum vitamin A is generally decreased in all types of AILD. Vitamin A is mainly stored in the liver. For patients with cholestasis, the major circulating primary BAs and formation of sulfate‐conjugated BAs are abnormally increased, then BAs required for physiological metabolism in the gut are relatively insufficient. It impairs intestinal uptake of fat‐soluble vitamins, including vitamin A, resulting in less retinol stored in the liver and thus less retinol released into the blood. Bile duct injury was found in both PBC and PSC, which was histologically characterized by cholestasis. Therefore, it is not difficult to explain the decrease in serum vitamin A levels in PBC and PSC. For AIH, Pemberton et al. suggested that the decline in vitamin A levels might be associated with decreased vitamin A storage capacity of impaired hepatocytes and activated HSCs.^21^ In the liver, vitamin A is involved in many important physiological functions. Vitamin A and its metabolites activate retinoic acid receptor (RAR) and retinoid X receptor (RXR) to generate RAR/RXR heterodimers and regulate BA synthesis and metabolism.^54^ In addition, it has been shown that vitamin A affects the expression of fibroblast growth factor 19 (FGF19), and then inhibits the expression of CYP7A1, which is a rate‐limiting enzyme of bile salt synthesis.^55^ Vitamin A supplementation may be beneficial for inducing bile excretion, alleviating cholestasis, and inhibiting HSC activation. However, excessive intake of vitamin A leads to acute and chronic toxic reactions, even causes cirrhosis and veno occlusive disease.^56^ Thus, the drug dosage of vitamin A needed to be further studied.

B vitamins are essential for promoting metabolism in the liver by converting sugars, fats and proteins into calories. Among them, vitamin B6, B9 and B12 are involved in methionine metabolism.^57^ Biagini et al.^45^ found a decrease in vitamin B6 and B9, particularly vitamin B9, which was considered to be associated with insufficient dietary intake and impairment of the folate enterohepatic circulation in PBC patients. Hcy is an important intermediate product of methionine and cysteine metabolism, and its metabolism is dependent on sufficient supplies of vitamin B9 and B12. These B vitamins deficiencies block the Hcy remethylation pathway, resulting in increased Hcy level.^58^ As a vascular damaging amino acid, Hcy is a risk factor for many diseases by regulating the coagulation and anticoagulation disfunction of endothelial cells, low density lipoprotein cholesterol oxidation, vascular sclerosis, inflammatory response, and abnormal metabolism of sulfur compounds.^59^ Amounts of evidence show that Hcy level is positively correlated with the severity of liver injury,^17^, ^18^ which is also supported by our conclusion.

Evidence have shown that vitamin C improves liver damage, possibly by suppressing inflammatory response and oxidative damage.^60^, ^61^ Our study found that vitamin C was decreased only in patients with PBC, but did not differ in patients with AIH. But the exact reason is not clear. In addition, with limited involved studies, such conclusion may not be very reliable.

Vitamin D plays a role in the body metabolism in different forms. Neither endogenous cholecalciferol converted by 7‐dehydrocholesterol stored in human epidermal keratinocytes, nor vitamin D2 and D3 ingested from food, are biologically active. They must work through converting to 25(OH)D2 and 25(OH)D3 by the 25‐hydroxylase system in the hepatocyte microsomes and then to 1,25(OH)2D3 under catalysis of the 25‐hydroxyvitamin D‐1 alpha‐hydroxylase in the proximal convoluted tubules of the kidney.^62^ Both 25(OH)D2 and 25(OH)D3 are collectively known as 25(OH)D, but 25(OH)D2 content is relatively low and difficult to be distinguished. Compared with 25(OH)D3, 1,25(OH)2D3 possesses the strongest activity against rickets, and the role of its metabolites in regulating calcium and phosphorus is higher than 25(OH)D3 200 times.^63^ Nevertheless, 1,25(OH)2D3 is homeostatic regulated by serum calcium and phosphorus concentration, parathyroid hormone, as well as calcitonin, and it has a short half‐life (4‐8 h).^63^ Thus, 25(OH)D3 is the main storage form and commonly used to reflect the nutritional status of vitamin D in the body. Since 25‐hydroxylase is mainly present in the hepatocyte microsomes, vitamin D metabolism is closely related to liver diseases. Vitamin D receptor has been found on the surface of almost all cells of the immune system, which may partly explain why abnormal vitamin D metabolism affect the development of some autoimmune diseases. It has been demonstrated that the polymorphisms of vitamin D receptor gene are significantly associated with increased individual susceptibility to develop chronic AILD.^64^, ^65^ For PBC, lacking bile salts affects the absorption of fat‐soluble vitamins and fat digestion, which may be one of the causes of vitamin D deficiency. AILD tends to occur in female. Compared with men, female are more likely to suffer from vitamin D deficiency due to the enhancement of vitamin D receptor gene expression and transcription by estrogen.^66^ In the PSC, vitamin D could reverse CD28‐T cells mediated inflammatory response, thereby reducing bile ducts damage caused by high levels of TNF*α* and IFN*γ* cytokines.^67^ The degree of vitamin D deficiency is positively correlated with the severity and progression of chronic liver disease. Paternostro et al.^68^ found that when vitamin D ≤ 10 ng/ml, the risk of adverse events, such as ascites, portal hypertension and primary hepatocarcinoma as well as the mortality in patients with cirrhosis increased. Ebadi et al.^69^ also indicated that severe vitamin D deficiency was independently associated with a higher risk of developing liver cirrhosis (HR = 3.40, 95% CI: 1.30–8.87; *p* = .01) and liver‐related mortality or the need for liver transplantation (HR = 5.26, 95% CI 1.54–18.0; *p* = .008) in AIH. Vitamin D supplementation may improve vitamin D levels and reduce bone loss in patients with AILD,^70^ but the long‐term prognosis is uncertain because of lack of evidence.

The main active substance of vitamin E is alpha‐tocopherol, which is an important antioxidant against reactive oxygen species (ROS) in the body. Overloaded ROS can cause oxidative stress‐related damage, which is one of the pathogenic bases for chronicity and progression of liver diseases. Vitamin E deficiency reduces free radical scavenging, thus accelerating liver disease deterioration.^26^, ^61^ Moreover, vitamin E is involved in regulation of the expression of genes controlling cholesterol homeostasis, phospholipid metabolism, and lipid uptake.^71^ The vitamin E also plays a critical role in regulating the inflammatory response by inhibiting the expressions of IL‐1β, MCP and IL‐6, thus attenuating liver fibrosis.^72^ Some PBC patients with low serum vitamin E levels exhibit clinically significant psychomotor dysfunction.^13^ In addition, vitamin E supplementation contributes to inhibiting neutrophil chemotaxis and may improve digestive function in children with chronic cholestasis.^73^ Consistent with previous results, our study confirms that vitamin E deficiency is prevalent in AILD patients. But the jury is still out on how to supplement vitamin E in them, thus further research is necessarily needed.

There are two main forms of vitamin K: phytoformine or phylloquinone (vitamin K1) and menaquinone (vitamin K2). Vitamin K1 is primarily derived from the diet, while vitamin K2 is synthesized by the intestinal bacterial flora. Vitamin K1 can be converted to vitamin K2.^74^ As with other fat‐soluble vitamins, vitamin K deficiency is quite common in patients with cholestasis due to reduced BAs in the gut. And the decrease in vitamin K1 was accompanied by reductions in serum vitamin A and E levels in PBC patients, which may be partly attributed to decreased intestinal absorption. In addition, since patients with liver diseases are prone to peritonitis, the increased use of antibiotics can lead to a decrease in the production of vitamin K2 by the gastrointestinal flora.^75^ Vitamin K levels are closely related to the stage of liver disease, which is owing to a significant decrease in the ability of hepatocytes to synthesize coagulation factors in severe liver diseases. Therefore, vitamin K is recommended for the correction of prolonged PT and the prevention of bleeding in patients with cirrhosis or liver failure.^76^, ^77^ However, Strople et al.^31^ found that despite vitamin K supplementation, ongoing vitamin K deficiency was still common in cholestatic liver disease. Aldrich et al.^33^ reviewed amounts of studies and concluded that routine use of vitamin K had no benefit in the correction of cirrhosis‐related coagulation dysfunction. Therefore, the level of vitamin K in AILD and whether supplementation of vitamin K is worthwhile remains a matter of discussion and clinical practice.

This meta‐analysis has several strengths. Our study systematically explores the association of multiple vitamins and Hcy levels with AILD, with subgroup analyses as far as possible. Secondly, we have included domestic and foreign studies on the premise of quality assurance, which is relatively representative. In addition, our control groups were healthy individuals, so as to minimize the interference of other diseases.

However, these limitations should not be ignored in our meta‐analysis. First, the limited number of studies and small sample size of some studies may increase heterogeneity. Second, despite the subgroup analysis, the heterogeneity of some overall effect results is still high, and the possible sources of heterogeneity may include severe stages of AILD, gender, and so forth. It has been demonstrated that the level of 25(OH)D3 in peripheral blood of AIH patients in the active inflammatory stage was significantly lower than that in the remission stage.^36^ Besides, the mean level of 25(OH)D in advanced stage of PBC patients was lower than that in early stage patients.^38^ However, due to the limited data, it cannot be quantitatively analyzed. Third, there are no data on the association between other vitamins and AILD due to no studies that meet our requirements. Finally, some data are obtained from combination and transformation, and certain errors may be unavoidable. Therefore, more studies with large sample even RCT studies of vitamins supplementation, are needed in the future to verify the association between vitamins and AILD.

n conclusion, the current study shows that most vitamins seem to be deficient in AILD. Vitamins, like A, E, and 25(OH)D3, were decreased in both AIH and PBC/PSC, but vitamin C was decreased only in patients with PBC, not AIH. Vitamin B6, B9, K1, and 1,25(OH)2D3 were also significantly decreased in patients with PBC, but not supported by sufficient data. Serum vitamin B12 and Hcy levels were significantly elevated in them. Further clinical trials involving more patients are needed to verify these findings.

## AUTHOR CONTRIBUTIONS

The protocol of this meta‐analysis was designed by Jiahuan Li and Xiaoling Deng. Literature retrieval, data extraction and analysis were performed by Li Jiahuan and Shan Tian. Data confirmation and quality assessment of included studies were conducted by Bai Ci and Yuwen Xi. The manuscript was written by Li Jiahuan and revised by Xiaoling Deng. Any disagreement regarding the formation process of this meta‐analysis was resolved by consensus or adjudicated by Xiaoling Deng. All authors approved the final manuscript.

## CONFLICT OF INTEREST STATEMENT

None declared.

## Supporting information

Supporting information.

## Data Availability

The raw data supporting the conclusion of this article will be made available by the authors, without undue reservation.
