# Supplementary material for: Serum vitamins and homocysteine levels in autoimmune liver disease: A systematic review and meta‐analysis
Source: Immun Inflamm Dis. 2024 Apr 23;12(4):e1258. doi: 10.1002/iid3.1258 (PMC11037259; doi:10.1002/iid3.1258)

**Supplementary materials**

**Table S1.** Results of quality assessment using the Newcastle-Ottawa Scale for included studies.

| Study | Selection | | | |  | Comparability control for important factors |  | Exposure | | | Scores |
| --- | --- | --- | --- | --- | --- | --- | --- | --- | --- | --- | --- |
|  | Adequate definition of cases | Representativeness of cases | Selection of controls | Definition of controls |  |  |  | Ascertainment of exposure | Same method of ascertainment for cases and controls | Nonresponse rate |  |
| Huang Junjun, 2021 | * | * | * | * |  | ** |  | * | * | NA | 8 |
| Zhan Wang, 2020 | * | * | * | * |  | ** |  | * | * | NA | 8 |
| Sen Tao，2020 | * | * | * | * |  |  |  | * | * | NA | 6 |
| Zhang Li, 2018 | * | * | * | * |  | ** |  | * | * | NA | 8 |
| Lin Yan, 2018 | * | * | * | * |  | ** |  | * | * | NA | 8 |
| Zhang Guoyu, 2017 | * | * | * | * |  | ** |  | * | * | NA | 8 |
| Tian Erjun, 2017 | * | * | * | * |  | ** |  | * | * | NA | 8 |
| Li Weiwei, 2017 | * | * | * | * |  | ** |  | * | * | NA | 8 |
| Yang Weiming, 2015 | * | * | * | * |  | * |  | * | * | NA | 7 |
| Nancy Agmon-Levin，2015 | * | * | * | * |  | ** |  | * | * | NA | 8 |
| Cumali Efe，2014 | * | * | * | * |  | ** |  | * | * | NA | 8 |
| William J Cash, 2010 | * | * | * | * |  | ** |  | * | * | NA | 8 |
| Zhang Xingrong，2006 |  | * | * | * |  | ** |  | * | * | NA | 7 |
| Maria Rosa Biagini, 2006 | * | * | * | * |  | ** |  | * | * | NA | 8 |
| Annarosa Floreani，2005 | * | * | * | * |  | * |  | * | * | NA | 7 |
| Philip W Pemberton, 2004 | * | * | * | * |  |  |  | * | * | NA | 6 |
| Ali Aboutwerat, 2003 | * | * | * | * |  | ** |  | * | * | NA | 8 |
| A Verma, 2002 | * | * | * | * |  | ** |  | * | * | NA | 8 |
| A Floreani，2000 | * | * | * | * |  | ** |  | * | * | NA | 8 |
| Kris V. Kowdley, 1997 | * | * | * | * |  |  |  | * | * | NA | 6 |
| I Janczewska, 1995 | * | * | * | * |  |  |  | * | * | NA | 6 |
| A Nyberg，1988 | * | * | * | * |  | ** |  | * | * | NA | 8 |
| G P Jeffrey，1987 | * | * | * | * |  |  |  | * | * | NA | 6 |
| V Fonseca，1987 | * | * | * | * |  |  |  | * | * | NA | 6 |
| R J Sokol，1985 | * | * | * | * |  |  |  | * | * | NA | 6 |

NA, not available.

**Figure S1.** Forest plot of the meta-analysis on serum vitamin 25(OH)D levels in patients with PBC.


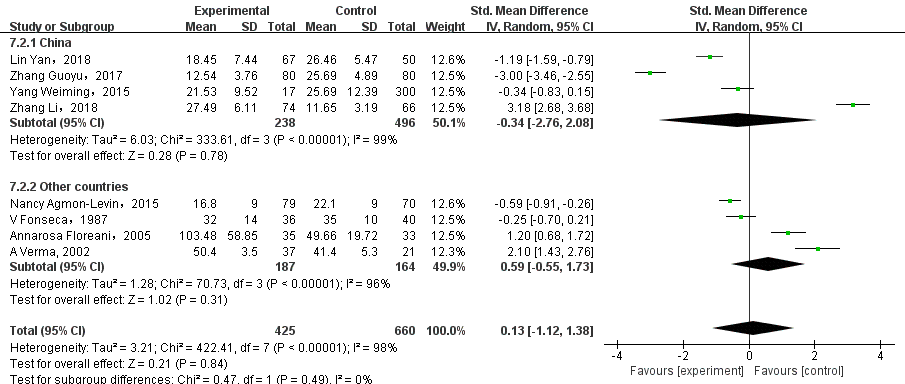

Supplement: Supplementary file 1 — Supporting information. [file IID3-12-e1258-s001.docx]
